# Variability in the Contribution of Different Life Stages to Population Growth as a Key Factor in the Invasion Success of *Pinus strobus*

**DOI:** 10.1371/journal.pone.0056953

**Published:** 2013-02-28

**Authors:** Zuzana Münzbergová, Věra Hadincová, Jan Wild, Jana Kindlmannová

**Affiliations:** 1 Institute of Botany, Academy of Sciences of the Czech Republic, Průhonice, Czech Republic; 2 Department of Botany, Faculty of Science, Charles University, Praha, Czech Republic; 3 Faculty of Environmental Sciences, Czech University of Life Sciences, Praha, Czech Republic; Helmholtz Centre for Environmental Research - UFZ, Germany

## Abstract

**Background:**

Despite the increasing number of studies attempting to model population growth in various organisms, we still know relatively little about the population dynamics of long-lived species that reproduce only in the later stages of their life cycle, such as trees. Predictions of the dynamics of these species are, however, urgently needed for planning management actions when species are either endangered or invasive. In long-lived species, a single management intervention may have consequences for several decades, and detailed knowledge of long-term performance can therefore elucidate possible outcomes during the management planning phase.

**Methodology and Principal Findings:**

We studied the population dynamics of an invasive tree species, *Pinus strobus*, in three habitat types represented by their position along the elevation gradient occupied by the species. In agreement with previous studies on the population dynamics of long-lived perennials, our results show that the survival of the largest trees exhibits the highest elasticity in all of the studied habitats. In contrast, life table response experiments (LTRE) analysis showed that different stages contribute the most to population growth rates in different habitats, with generative reproduction being more important in lower slopes and valley bottoms and survival being more important on rock tops and upper slopes.

**Conclusions:**

The results indicate that *P. strobus* exhibits different growth strategies in different habitats that result in similar population growth rates. We propose that this plasticity in growth strategies is a key factor in the invasion success of the white pine. In all of the investigated habitats, the population growth rates are above 1, indicating that the population of the species is still increasing and has the ability to spread and occupy a wide range of habitats.

## Introduction

Invasive species are known to have strong effects on native species, communities, and ecosystems, e.g., [1], [2], [3], also often having strong economic impacts, e.g., [4], [5]. All of these negative effects support the need for developing efficient strategies for their eradication [6]. Effective control of invasive species requires understanding the mechanisms allowing their populations to grow and spread. Such an understanding can be attained by developing models that combine information on local population dynamics with information on the distribution of suitable habitats for the species and its long distance dispersal [7], [6]. While this type of approach is an obvious way to understand the spread of invasive species, there are currently very few examples of such thorough analyses [6], [8], [9], [10]. The possibility of developing these models is clearly limited by the availability of good data on the demographic dynamics of species, particularly long lived ones. In recent years, there has been an increasing number of studies attempting to model population growth (e.g., [3], [11], [12], [13] and references therein) and to quantify the extent of variation in population dynamics using matrix models, e.g., [14], [15], [16], [17], [18]. These studies have generally demonstrated high spatial and temporal variation in population dynamics. However, only a few of these studies address alien species spreading to new areas, e.g., [19], [20], [21]. Additionally, most of the existing information regarding assessments of the importance of spatial and temporal variation for the vital rates of different developmental stages and for the growth of the resulting plant population comes from studies on short-lived herb species (e.g., [14], [15], [22]; also see Franco and Silvertown [23] for a list of plant species included in database of population projection matrices). However, good predictive models are even more important for long-lived species, such as many trees, whose management needs to be planned over decades. A similar under-representation of studies on the population dynamics of trees can be found in the literature on invasive species, which also mainly addresses herbs and shrubs e.g., [24], [25], [26]. Only a few models of invasive tree dynamics have been published [27], [21], [28].

Both gymnosperm and angiosperm tree species are known to be invasive, but angiosperms have been much more frequently addressed in the literature [29], [23]. Among gymnosperms, a particularly high percentage of invasive species has been reported in the *Pinaceae* family (12%, [30]). However, we are aware of only two studies investigating the population dynamics of an invasive *Pinaceae* species using matrix models (*Pinus nigra*, [21], [31]). This is surprising given that matrix population models enable us not only to assess whether the population of the species of interest is still growing but also to reveal the life cycle stages or transitions that are most important for its population growth and, thus, can help to focus future management measures on appropriate targets.

The strength of matrix population modeling has been shown by Ramula et al. [29]. These authors performed elasticity analyses of survival, growth and fecundity transitions in 21 invasive and 179 native species and revealed that invasive species are more sensitive to perturbations in their growth and fecundity (whereas survival during certain life stages was more important for populations of native species). However, for long-lived invaders, simulated reductions in either growth or fecundity alone were insufficient to achieve decline of the populations, and multiple reductions in the two types of transitions combined with survival were more effective. This finding reflects the typical situation for long-lived tree species; i.e., survival in later life stages is the most important transition [32], [33], [34]. Thus, it is evident that there are some general rules regarding the population dynamics of invasive plants and long-lived trees, but how these rules function together to determine the population dynamics of invasive tree species is not completely clear.

The aim of this study is to extend our knowledge of the population dynamics of invasive conifers and describe the spatial and temporal variation in the population dynamics of an invasive tree species, *Pinus strobus*, in a sandstone area in the Czech Republic, Europe. This species has been declared invasive, as it regenerates well in many habitats in central Europe [35], [36]. It also has the ability to spread over 1 km distances via seed dispersal [37]. To successfully address invasions of this species, it is necessary to understand the population dynamics of this species in all invasible environments over a sufficient number of years. We studied the species over multiple years as it is widely acknowledge that population dynamics strongly varies over time e.g., [15], [38], [39], [40], [41] and having data only from one time period can lead to largely misleading conclusions. In addition, we wanted to describe population dynamics in multiple locations because habitat conditions often play a crucial role in determining population dynamics of a species e.g., [15], [39]. Detailed knowledge of both temporal and spatial variation in population dynamics is thus necessary to correctly understand the potential of species to increase the local populations and to spread in a landscape.

To do this, we studied *P. strobus* in three different habitat types representing the 3 major environments in our study region and compared results regarding the population dynamics of this species based on detailed data collected over 3 transition intervals and additional rough data available for 11 transition intervals. We sought to determine whether its populations were still increasing and to identify spatio-temporal variability in its population growth rate. Specifically, we asked the following questions. (1) What is the spatial and temporal variation in the growth and mortality of these trees? (2) What is the spatial and temporal variation in the local population dynamics of the species? (3) Which vital rates contribute the most to the population growth rate? (4) What is the difference in the predicted population performance when examining a detailed 3-year dataset compared to a simplified 11-year dataset?

To achieve these aims, we collected detailed data on the complete life cycle of the species with a focus on seedling and sapling stages in three habitat types representing different positions along the slope (referred to as habitat types in the subsequent text) over three transition intervals. We selected the position along the slope as the studied gradient, as it has a major effect on the vegetation in the area [42], and we expected that it would also have a major effect on the dynamics of *P. strobus*. To obtain better insight into the temporal variation in the growth of this species, we also collected data on the size increments of the trees over 11 transition intervals using a backward measurement method and employed this information to create matrix models for an 11-year period. The clear advantage of the 3-year dataset is that we have detailed data on all life cycle transitions from each year studied and these data thus correspond to data commonly presented in various studies on plant demography [15], [38], [39]. However, a common critique of field collected demographic data is that they cover a low number of years and thus could miss important climatic events that may be decisive for long term dynamics of the species. We thus used the advantage of the fact that in *P. strobus* it is possible to determine past growth based on growth increments and created a dataset describing growth of the tree over 11-years. Thanks to this we thus had higher chance to capture these extreme events. On the other hand, we did not have detailed data of generative reproduction from each year for this dataset and thus had to use population means.

## Methods

All the field work was possible thanks to a collaborative agreement between the authors (lead by V. Hadincová) and the administration of the National Park (lead by Z. Patzelt) no. 2437/05/MaJ/KL.

### Study Species

*Pinus strobus* L. (the eastern white pine, also known as the northern white pine) is native to eastern North America [43]. In the studied area in the Czech Republic in Europe, this species has been cultivated since the end of the 18^th^ century. It was introduced into mixed conifer forests to improve the species composition and to prevent pest development in species-poor forests on nutrient poor sandy soils and sandy loams [44]. The species’ ability to regenerate was noted during the early days of its cultivation [44], [45], but its spread was not detected until the 1990 s, when it entered predominantly acidophilous pine (*Dicrano-Pinetum*) and oak-pine (*Vaccinio vitis-idaeae-Quercetum*) forests. *Pinus strobus* is now a component not only of cultivated mixed forests but also of other forest types and is found among sparse vegetation on rocks. It mainly competes with two native, principally forest trees, *Pinus sylvestris* and *Picea abies,* both in the canopy (the average height of trees more than 80 years old in the area is 21 m for *Pinus sylvestris*, 24 m for *P. strobus* and 25 m for *Picea abies*) and in the undergrowth because of abundant regeneration. The abundant self-sown trees can suppress not only native tree regeneration but also the growth of mosses and herbs in the undergrowth [36]. The species is currently being managed in different parts of the study area. However, the studied localities were selected in areas where there are no management measures suppressing *P. strobus*, and our results are therefore not affected by any management actions.

### Study Area

The study was conducted in the Bohemian Switzerland National Park, which is part of the Elbe-river Sandstones in the Czech Republic, Central Europe. The study area is a sandstone erosion landscape extending along the Elbe valley on the Czech side of the German-Czech border. The region is characterized by a temperate climate with mean annual temperatures of 6–7.5°C and precipitation of 700–850 mm. The precipitation is evenly distributed throughout the year, while the temperature displays seasonality, with snowy period occurring from December to February and a warm period from June to August [46]. The area is primarily covered with woodlands and is characterized by frequent alternation of acidic sandstone rocks, plateaus, deep canyon-like valleys and gorges and, occasionally, basalt outcrops. Numerous combinations of different micro- and meso-climatic conditions result from sharp gradients between dry, windy, insulated rock tops with shallow soil and wet, shadowy, cold bottoms of gorges. Mesic habitats also occur between these two extremes and are usually situated along slopes under cliffs. These sites have deeper soils, favorable soil humidity, and are not exposed to direct sun and strong wind [47].

**Figure 1:**
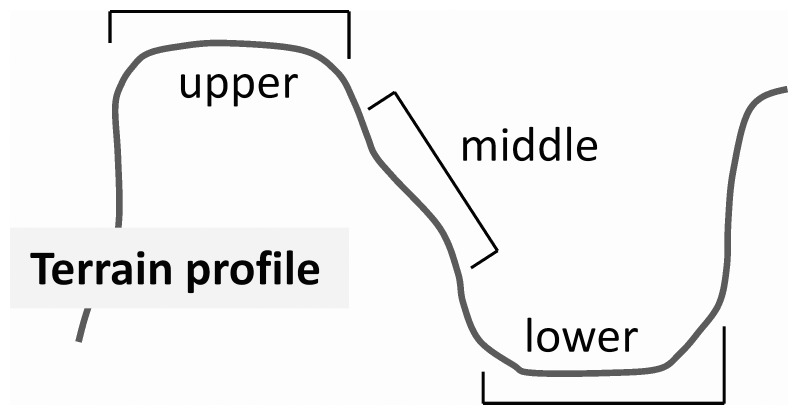
Schematic diagram depicting position of the upper, middle and lower habitat types within a locality.

**Figure 2:**
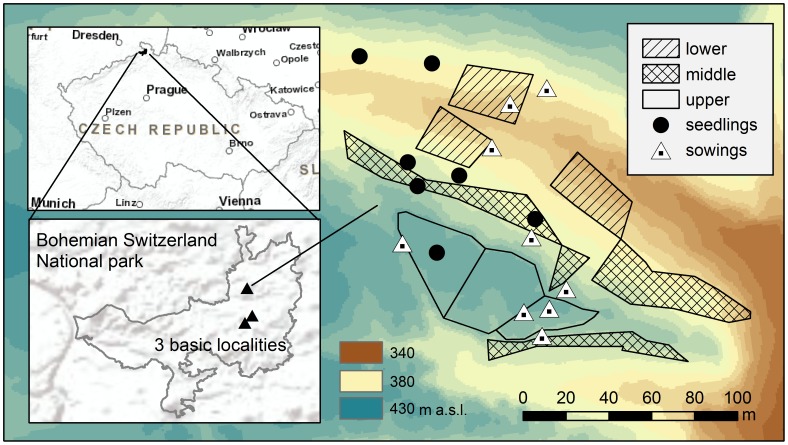
Map showing the position of the study area, the 3 basic study localities and the specific plots within a single locality. Background for basic topographic maps were provided by ESRI and digital elevation model by TU Dresden.

For the purpose of the study, we selected three different basic localities 1.2–3.2 km apart. All of the localities are situated between 50°51′17″ N and 50°54′27″ N and 14°24′7″ E and 14°25′48″ E. Within each locality, three study sites were established, which were situated in three different habitat types, with the habitat type being defined by the position along the slope as follows: (i) upper positions usually including also rock edges; (ii) middle positions; and (iii) lower positions represented by lower slopes and ravine bottoms (Fig. 1, Fig. 2). We studied population dynamics in these three types of habitat because the position along the slope has been shown to have a major effect on vegetation in the area [42]. The slope aspect would also be expected to be ecologically important in many other areas. However, in the study area, where there are deep gorges, the aspect is highly variable. The relief and local supply of water, microelements, and solar radiation are therefore more important for shaping environmental conditions in this region [42].

In the study area, *P. strobus* trees were planted 100 years ago, and no additional planting occurred after this date. As a result, the largest of the studied trees are of plantation origin, whereas all of the other trees were produced through natural regeneration.

### Size Categories

To allow analysis of the results using stage-based transition matrices, we divided the trees into nine size categories: (1) seedlings, (2) non-seedlings <0.15 m, trees between (3) 0.150.5 m, (4) 0.51 m, (5) 1–2 m, (6) 2–8 m, and (7) 8–16 m, and (9) trees >16 m. The first size category includes first-year seedlings, which are distinct from older individuals and often suffer much higher mortality. The second category includes individuals up to 0.15 m tall. At this size, they are already relatively well established, and their mortality declines strongly. Based on previous observations, individuals within these size categories generally exhibit similar performance. Within each locality and habitat type, 30 individuals per size category were recorded in each transition interval cf. [48]. The 30 individuals were chosen within 3 subplots, with 10 individuals in each subplot. The subplots within localities were selected randomly in areas where trees of the given size category were found at distances of between 25 and 100 m from each other. All individuals were labeled to allow easy identification of the trees over time. This species does not have a permanent seed bank [43], and seeds were therefore not included as a separate size category in our models.

### Individual Growth

To estimate the growth of individual trees, we measured the distances between subsequent whorls on trees greater than 0.15 m tall. These distances represent yearly increments in the growth of this species [49], [37]. This method was suitable because increment size does not change after the growth season, and growth irregularities such as lammas and/or proleptic shoots [50] were found very rarely. For trees of less than 8 m in height, the increments were measured on living trees using a tape measure; larger trees were cut prior to being measured. Measurement of the increments on the large trees was performed at the end of the study period to ensure that the experimental plots were not disturbed by tree cutting. For each tree, we also recorded the total height and age by counting the number of increments [37]. The oldest measured increment was from 1950. Because we had too few increments in some size categories from 1950 to 1996, these data were removed from the analyses, and only increments from 1996 onwards (i.e., from the last 11 years) were used (1, 372 trees and 13,500 increments in total). All of the measured increment sizes are presented in Figure S1. All of the increments on all living trees were measured in October 2007. However, the 2006 increments were the last increments used in the analyses to ensure that we had recorded the final length of all increments cf. [51].

Data on the growth of trees less than 0.15 m in height were obtained from individual measurement of seedlings marked in experimental sowing plots between 2004 and 2006 and at seedling plots (see *"Generative reproduction"* below) in a total of 537 individuals. These measurements were conducted in every year in October.

### Mortality

The mortality of trees up to 0.15 m in height was estimated from the individual monitoring of seedlings marked in experimental sowing plots between 2004 and 2006, as described above. To estimate the mortality of trees from size categories greater than 0.15 m up to 8 m, we counted the number of dead trees in each size category in each locality and habitat type. For this purpose, we marked all of the dead trees of a given size category in each plot at the end of 2003. The size of the plot was selected to include at least 30 trees in the largest size category in each locality and habitat type and ranged from 36 to 1670 m^2^. This high variation in size and thus in density of mature *P. strobus* was given by the fact that the mature *P. strobus* trees are mostly of plantation origin and they were planted in different manner in different locations. All these plots were, however, located in dense forest stands. The locations with low density of *P. strobus* had high density of *P. abies* and/or *P. sylvestris* and possible intra-specific competition between *P. strobus* was at these sites replaced by inter-specific competition. From 2004 to 2006, we recorded all new dead trees annually (at the end of the year) and assigned them to a size category. The process of tree decomposition takes longer than one year, and thus, all of the dead trees should have been observable (pers. obs.). Only trees with no green needles were considered to be dead. We also recorded the number of living trees in a given size category in the same plots each year.

Estimating the mortality of trees greater than 8 m was more difficult because mortality is quite rare in these trees, and trees of this size are sparse. On the other hand, dead individuals of these large trees can be found for longer periods of time. We therefore recorded all of the trees that died between 1 and 10 years ago (i.e., between 1997 and 2006) in larger plots in each habitat type. Because of the extremely low number of trees of greater than 8 m at some localities, we sampled a wider range of localities, separated by habitat type. These localities corresponded to the localities of the seedling plots (see below). Distinguishing trees that died approximately 1 to 10 years ago is possible if only trees that are still standing are counted. Trees that died more than 10 years ago have usually already fallen. Although some trees that have been dead for more than 10 years could be still standing, they can easily be recognized because they only have bark at the bottom of the trunk and do not have fine branches. These rules for determining the age of dead trees were based on personal experience with trees observed in other plots in the same area between 1998 and 2007 (Hadincová unpubl.). Because the estimation of the age of the dead trees is not exact, we explored the effect of changing the age range of dead trees to between 1 and 8 or 1 and 12 years old, which only had a minor effect (max 1% change in the population growth rate) on the results, and these alternate ranges were therefore not considered further. Finding all of the dead trees that died in the last 10 years was also made possible because the study area has been under special management since 1972. Since that time, the forest management policy has been extensive, and no trees (alive or dead) have been removed from the study area. We also counted all living trees greater than 8 m in height in the same areas. In all cases, we divided the trees into the categories of greater than 8 m and greater than 16 m in height. The ratio of living to dead trees per year served as an estimate of the mortality per year in all cases (for trees above 8 m, the number of dead trees must be divided by 10, which was the number of years for which data were collected).

### Generative Reproduction

Seedling germination was assessed with two different approaches in sowing plots and seedling plots. First, we established 15 sowing plots (0.25 m×0.25 m) and 15 control plots in 2003, 2004 and 2005 in each habitat type and locality and also at two additional nearby localities to increase our sample size for the extremely variable early stages of plant growth (i.e., 45 sowing plots per habitat type (3 types) and locality (5 localities), for a total of 675 sowing plots plus the same number of control plots). One hundred and twenty seeds were sown per sowing plot in autumn, i.e., at the time of *P. strobus* seed release, in the year of establishment of the plot (2003, 2004 or 2005). The number of seedlings that appeared was observed in the following autumn. No seedlings were detected in the control plots, and they are therefore not mentioned further. The seedlings were marked, and their growth increments and mortality were measured in the two subsequent years. Because of extremely high variation among the single plots and no significant effect of position on the transition from new seedlings to saplings <0.15 m, but a strong effect of year on this transition, we combined the data from the three positions to estimate the transition probability from seedlings to small saplings.

Second, we estimated seedling regeneration by counting the number of 2-year-old *P. strobus* seedlings in plots with a known number of reproductive trees (seedling plots). We assume that only the trees greater than 16 m in height are reproductive for the purpose of this analysis. This assumption is reasonable because trees between 8 and 16 m produce only 6, 1 and 9% of the number of seeds produced by trees above 16 m in the upper, middle and lower positions, respectively (Hadincová, unpubl.), and inclusion of these values in the transition matrices would only change the population growth rates by 0.04, 0.01 and 0.12% in the upper, middle and lower positions, respectively.

We counted 2-year-old and not 1-year-old seedlings (which are a necessary part of our transition matrices) because 1-year-old seedlings are very difficult to find in larger plots, and their numbers are highly dependent on the exact timing of counting because of high first year mortality. Seedlings were counted in three subsequent years (2005–2007). These seedlings originated from seeds produced in 2003, 2004 or 2005, which were used in the sowing experiments. In each year, we selected 3 to 9 large plots in each habitat type. These plots were situated in the three basic plots and in their close vicinity. The seedlings were counted in a total area of 2300 to 4900 m^2^ per year in each habitat type by slowly walking in a grid over the whole area. The total number of seedlings was divided by the area, and the number of seedlings per 1 m^2^ was estimated. The number of mature trees (the expected sources of the seedlings) was counted in an area approximately three times as large to take into account the fact that the seeds do not fall directly under the parental trees and that mature trees are not evenly distributed in the forest. The number of mature trees was divided by the size of the surveyed area to estimate the number of adult trees per 1 m^2^. By dividing the number of seedlings per 1 m^2^ by the number of adult trees per 1 m^2^, we obtained information on the number of seedlings per adult tree. The seedling plots were situated in the center of the plots used to count the mature trees. To estimate the number of 1-year-old seedlings per mature tree, we divided the estimated number of 2-year-old seedlings per mature tree by the mortality of 1-year-old seedlings estimated in the sowing experiment above. This information was necessary as number of 1-year-old seedlings per mature tree represented transition from mature trees to 1-year-old seedlings (stage 1) and was thus part of our transition matrices (see *Size categories* section above).

When estimating the number of seedlings per mature tree, we need to assume that the export of seeds from trees growing in the plot to outside of the plot equals the import of seeds into the plot from outside. We expect that this assumption is reasonable, as we counted mature trees in larger areas than seedlings and therefore obtained robust information on the mean mature tree densities in the areas.

In the seedling plots, we also sampled 166 seedlings that were at least 2 years old but were smaller than 15 cm and measured the length of their increment. These values complemented the data on the growth of trees smaller than 15 cm from the sowing plots.

### Construction of Transition Matrices

Estimates of transition probabilities for individuals between different size categories are commonly derived from data on marked individuals that are followed over time e.g., [52], [16], [53], [54]. However, in a slow-growing species such as *P. strobus*, this approach is not practical, as individuals could remain in one size category for many years in some cases. Thus, the transition probabilities are very sensitive to the exact initial size of the marked individuals. To avoid this problem, we estimated the transition probabilities for individuals between different size categories using data on the increments of trees in each size category. The probability of transitioning into the subsequent size category was expressed as
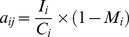
(1)where I_i_ is the mean increment length in size category i; C_i_ is the width of size category i (the difference between the upper and lower limit of the size category); M_i_ is the mortality in size category i; j = i–1, i.e., the size category preceding size category I; and a_ij_ gives the probability that an individual in size category j at time t moves to size category i at time t+1 [55]. The probability of stasis was expressed as



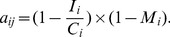
(2)Using this approach, we were able to estimate the transition probabilities between size categories, without needing to follow the growth of the trees over many years. The data on vegetative transitions were combined with the data on generative reproduction and seedling survival described above.

To explore the spatial and temporal variation in population dynamics, we constructed transition matrices for each combination of year and habitat type. We constructed two sets of year-specific matrices. First, we constructed transition matrices for three transition periods between 2004 and 2007. In this case, all of the matrix elements were based on year-specific data. Because not all of the data on the reproduction and mortality of large trees came from the same localities as the other data, and the sample sizes within each locality were relatively small (max. of 30 trees per locality, habitat type and size category), we always combined data from a single habitat type over the three localities.

Second, we constructed transition matrices for each transition period between 1996 and 2007 using the data on the increment length in each year. All of the other matrix elements (mortality, generative reproduction) were held constant between years in this case and were based on the mean of the data from 2004 to 2006.

Using this approach, we obtained two different datasets. The first dataset includes more detailed data, but from only 3 transition intervals. The second dataset comes from 11 transition intervals with constant mortality and generative reproduction. This dataset covers a longer time period, but the data are less detailed.

### Data Analysis

We employed analysis of variance (ANOVA) to analyze the effects of the locality, habitat type, year of the increment, size category, and age on the lengths of the increments in the trees from categories between 1 and 16 m in height from 1996 to 2006. To take into account the fact that the increments within a single tree were not independent, the effect of locality and habitat type was tested against the effect of the tree code, instead of against the residual variance.

We applied generalized linear models with a Poisson distribution to analyze the effects of the size category, year, locality, habitat type and their interactions on the number of dead trees from 0.15 m up to 8 m in height from 2004 to 2006. The number of living trees in the same area was employed as a covariate to account for differences in plot size and tree density between localities and habitat types.

Demographic data were analyzed using transition matrix models. The transition matrix represents the quantitative demographic data that describe the life cycle of a population with a size category structure in a standard format [55]. A matrix population model has the general form.
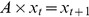
(3)and describes the dynamics of a population comprised of i ( = j) size categories. **A** is a transition matrix with i rows and j columns, containing matrix elements, a_ij_, which define transitions from population size category j to size category i in a predefined time interval (t to t+1), and **x**_t_ is a column vector containing the number of individuals in each size category at time t. The transition interval corresponds to 1 year in all cases.

Analysis of a projection matrix yields a finite rate of increase, λ, for the population and generates information on changes in the population growth rate, δλ, following a small change in a_ij_ (δa_ij_), the sensitivity. We used proportional sensitivity, or elasticity, as a measure of the contribution of a matrix element to fitness. High elasticity indicates that a small proportional change in a given matrix element leads to a large change in the population growth rate [56].

Each transition probability estimate and therefore each estimate of the population growth rate and of elasticity is confined with an error because of the limited number of individuals that can be sampled. To estimate this error, we calculated bootstrap confidence intervals [57] for the growth rate and elasticity for each transition matrix (from each population and year). This was achieved by bootstrapping the original data employed to derive the transition matrices 10 000 times. Based on the results, we constructed 95% confidence intervals of population growth rates for each population and year [58] using our MATLAB script [59], [54], [60].

To summarize the data on population growth rates over habitat types and years, we used a stochastic simulation approach as suggested by Caswell [55] and Rydgren et al. [61]. For each set of matrices, we drew a sequence of matrices. Each matrix from the set was drawn at random and with an equal probability of a matrix being selected, and we simulated population growth using this matrix sequence. Each simulation was performed for 10 000 one-year intervals. The simulations were conducted using a MATLAB script developed in a previous study [53]. We ran these stochastic simulations for the original matrices as well as for the above-described bootstrapped matrices. By running the stochastic simulations for the bootstrapped matrices, we obtained the 95% confidence interval of the stochastic population growth rate.

We also employed the transition matrices to calculate the stable stage distribution for each habitat type. The stable stage distribution provides information on proportion of individuals in each size class under the assumption that population dynamics are stable over a longer period of time [55].

To identify the contribution of the habitat type and year to the overall population growth rate and the contribution of each single transition to this population growth rate, we performed a life table response experiment. Specifically, we used a two-way life table response experiment (LTRE) with a fixed factorial design. An LTRE is a form of retrospective analysis that allows quantification of the factors responsible for the observed variation in population growth rates [55], (Text S1). For each factor, the contributions to population growth rate of all the levels of the given factor sum to zero. As a result, positive contribution means that the transition increases the overall population growth at the given level of the factor, while negative contribution means that the transition decreases the overall population growth at the given level of the factor (Text S1). The negative values of contribution thus indicate that this specific transition represents a weak point in the life cycle at the given level of the factor.

## Results

Over the course of the study, we followed the fate of over 800 individuals in all of the three main habitat types in the study area. Furthermore, we measured the increment length in an additional 1372 trees to obtain more detailed insights into changes in growth over a longer period of time. For some of the size classes, we obtained information on growth for as long as 47 years (Figure S1).

### Individual Growth

Tree growth (i.e., the lengths of increments) was significantly affected by the locality, habitat type, year, size category and tree age (Table 1). Several interaction terms were also significant (Table 1). However, the only variables that substantially contributed to explaining the variation in tree growth were the size category and year of creation of the increment (explaining 26% and 9% of total variation, respectively). Tree age explained less than 1% of the variability in increment length. The lengths of the increments within the size categories clearly declined over the last 11 years, indicating that the individual vigor of the trees was declining with time (Figure S2). However, data from longer time periods available for particular size categories suggest declines were not occurring in all cases and that the sizes of the increments varied greatly over time, including periods of decreases as well as increases (Figure S1).

**Table 1:** Effect of the locality (geographical locations of plots, each with all 3 habitat types), age of the tree in the time when the increment was created, habitat type (position on the slope), year in which the increment was created, size category of the tree in the year in which the increment was created, and their interactions on the lengths of increments in *P. strobus* analyzed using ANOVA.

	Df	F	p	R^2^
Locality	2	107.3	< 0.001	0.006
Age	1	253.3	< 0.001	0.007
Habitat type	2	116.2	< 0.001	0.007
Year	10	71.2	< 0.001	0.090
Size category	5	1859.6	< 0.001	0.261
Locality×age	2	4.7	0.009	0.001
Locality×habitat type	4	85.7	< 0.001	0.028
Locality×year	20	5.3	< 0.001	0.009
Locality×size cat.	10	9.8	< 0.001	0.008
Age×habitat type	2	2.1	0.128	–
Age×year	10	4.9	< 0.001	0.006
Age×size category	5	110.1	< 0.001	0.044
Habitat type×year	20	1.1	0.280	–
Habitat type×size cat.	10	2.8	0.002	0.001
Year×size cat.	212	2.4	< 0.001	0.014
Locality×age×habitat type	4	13.7	< 0.001	0.004
Locality×age×year	20	0.7	0.813	–
Locality×age×size cat.	10	2.9	0.001	0.002
Locality×habitat type×year	40	4.5	< 0.001	0.015
Locality×habitat type×size cat.	19	6.8	< 0.001	0.010
Locality×year×size cat.	92	1.0	0.392	–
Age×habitat type×year	90	0.7	0.972	–
Age×habitat type×size cat.	10	2.3	0.010	0.002
Habitat type×year×size cat.	370	0.8	0.997	–

No four-fold or higher interactions were significant, and these interactions are therefore not shown. Df Error = 1360.

The number of dead trees depended significantly on all of the individual predictors (Table 2). Only the size category and year of death, however, had relatively high explanatory power, explaining 14% and 33% of the total variation in the dataset, respectively. Mortality was the highest in the 1^st^ transition interval and the lowest in the 2^nd^ transition interval.

**Table 2:** Effect of the size category, year of death, locality, habitat type (position on the slope) and their interactions on the number of dead trees assessed using generalized linear models with a Poisson distribution.

	DF	Resid. DF	P	R^2^
Number of living trees	1	304	< 0.001	0.02
Size category	4	300	< 0.001	0.14
Year	2	298	< 0.001	0.33
Locality	2	296	< 0.001	0.02
Habitat type	2	294	< 0.001	0.07
Size category×year	8	286	0.091	–
Size category×locality	8	278	0.656	–
Size cat.×habitat type	8	266	0.031	0.01
Year×locality	4	274	< 0.001	0.02
Year×habitat type	4	262	< 0.001	0.02
Locality×habitat type	4	258	< 0.001	0.07
Size cat.×year×locality	16	242	0.609	–
Size cat.×year×habitat type	16	226	0.128	0.02
Size cat.×locality×habitat t.	16	210	0.001	0.03
Year×locality×habitat type	8	202	< 0.001	0.02

The number of living trees in the observed plot was used as a covariate to account for differences in the plot size and tree density between plots and is therefore not included in any interaction term. No four-fold or higher interactions were significant, and these interactions are therefore not shown.

In contrast to the size category and year, habitat only had a small effect on both increment length and mortality, explaining less than 1% of the total variation (Table 1; Table 2).

### Population Growth

**Figure 3:**
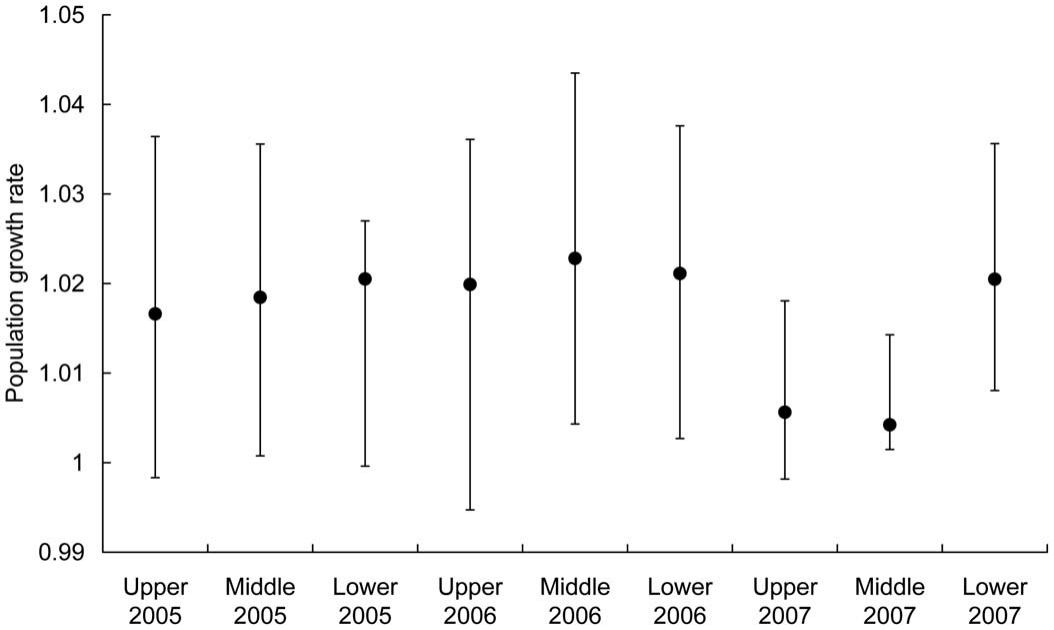
Effect of the year and habitat type on the population growth rate. Effect of the year (2005–2007) and habitat type (position on the slope – upper, middle or lower) on the population growth rate determined using matrices for each habitat type and transition interval. The calculation is performed using the 3-year dataset containing year-specific data for all stages of the life cycle. Mean ±95% confidence interval.

**Figure 4:**
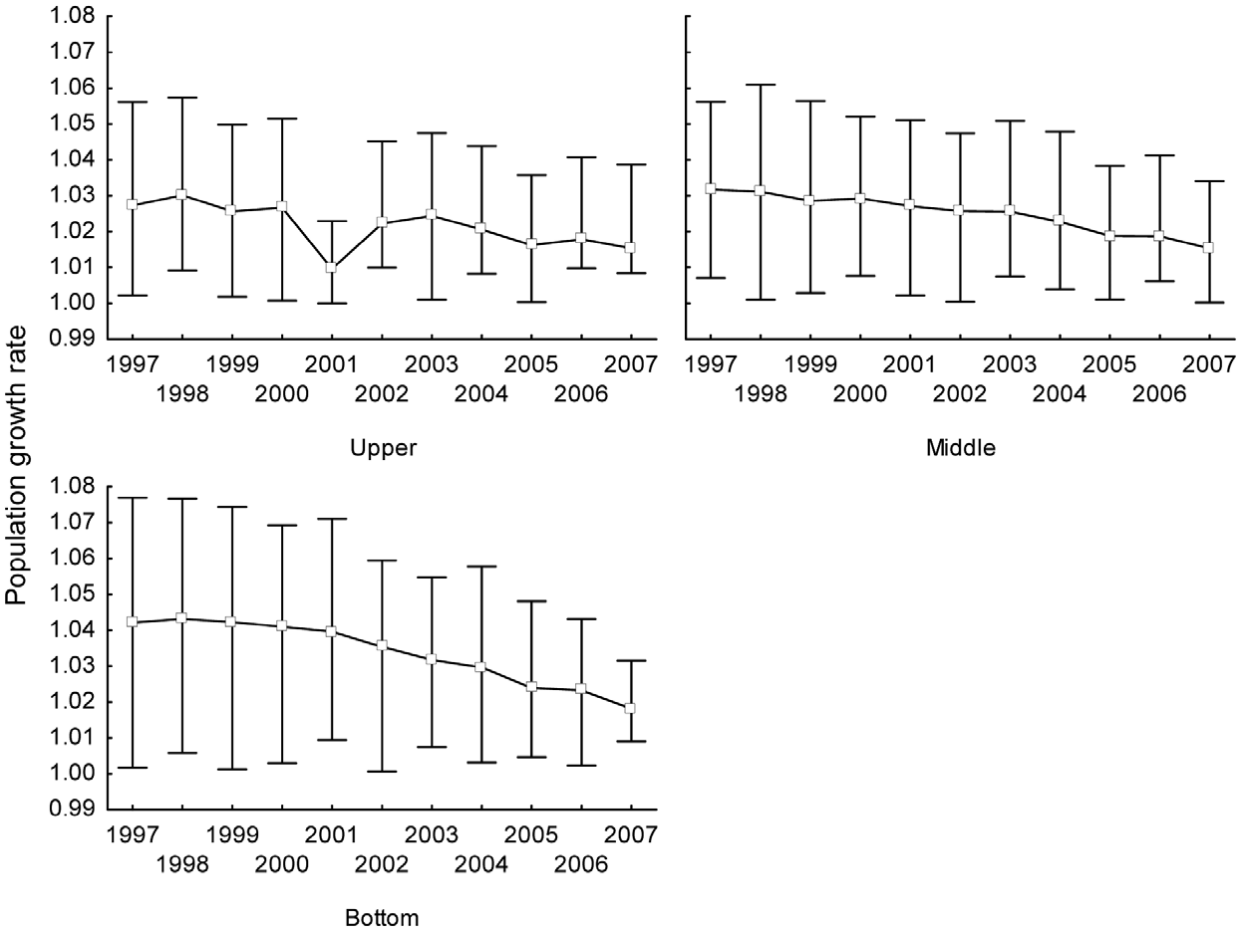
Population growth rates in the 3 habitat types. Population growth rates in the 3 habitat types (position on the slope – upper, middle or lower) over 11 years (1997–2007). The mortality, natality and growth and survival of trees up to 0.5 m are kept constant in these matrices. Mean ±95% confidence interval.

The population growth rates based on single transition matrices (Table S1) constructed separately for the three transition intervals for each habitat type ranged between 1.005 and 1.022, with largely overlapping confidence intervals (Fig. 3). Similar patterns could be observed in the matrices from the 11 transition intervals (Fig. 4, between 1.01 and 1.042). In this case, there was a visible trend of declining population growth rates over time, corresponding to the decline in the lengths of the increments. The lowest and most variable population growth rate was observed at the upper position.

When the transition matrices were combined over the transition intervals within each habitat type using the stochastic simulation approach, the population growth rate was found to be highest at the middle position (1.080, 95% conf. int. 1.062–1.110), followed by the lower position (1.042, 95% conf. int. 1.031–1.058) and the upper position (1.024, 95% conf. int. 1.022–1.037) in the 3-year dataset. A similar pattern with even smaller differences between habitat types was observed in the 11-year dataset (population growth rates corresponding to 1.035, 1.025 and 1.024 at the lower, middle and upper position, respectively). The long-term population growth rate combining all of the matrices included in the 3-year dataset (i.e., over all habitat types and transition intervals) was 1.077 (95% conf. int. 1.070–1.097). This value was comparable to the value obtained from the 11-year dataset, which yielded a population growth rate of 1.063 (1.061–1.082).

In spite of the limited differences in population growth rates observed between habitats and years, both habitat types and years differed strongly in the stable stage distribution (Table 3). Most importantly, the populations in the middle positions should present approximately 56% seedlings under stable population dynamics, and populations in the lower positions should exhibit 30% seedlings under stable population dynamics. In both of these habitat types, over 70% of all plants belonged to the two smallest size categories. In contrast, the populations on the upper areas of rocks are predicted to contain only 8% seedlings under stable population dynamics and a larger number of trees in stages 2 (up to 0.15 m), 3 (0.16–0.50 m) and 7 (4–8 m). These differences partly correspond to the differences observed in the field, with a large number of seedlings and small saplings (up to 0.5 m) being found in lower and middle positions and a relatively small number of individuals of small stages and higher number of individuals of larger stages observed at the upper positions (Table 3).

**Table 3:** Observed and stable stage distributions in the different habitat types (upper, middle and lower), mortality and individual tree density based on the 3-year dataset.

	Observed stage distribution(proportion of individuals ina given stage from the wholepopulation)			Stable stage distribution(expected proportion ofindividuals in a given stagefrom the whole population)			Individual density(individuals/m^2^)			Mortality (proportionof dead trees)		
	Upper	Middle	Lower	Upper	Middle	Lower	Upper	Middle	Lower	Upper	Middle	Lower
Seedlings	0.18	0.50	0.49	0.08	0.56	0.3	0.07	0.46	0.57	0.473	0.473	0.473
Up to 0.15 m	0.09	0.24	0.23	0.32	0.26	0.42	0.03	0.22	0.27	0.295	0.677	0.545
0.15–0.5 m	0.13	0.13	0.14	0.12	0.05	0.12	0.05	0.12	0.16	0.017	0.010	0.018
0.5–1 m	0.17	0.03	0.04	0.09	0.03	0.07	0.06	0.03	0.04	0.022	0.061	0.089
1–2 m	0.16	0.02	0.04	0.08	0.02	0.03	0.06	0.02	0.04	0.043	0.094	0.123
2–4 m	0.12	0.02	0.03	0.08	0.02	0.02	0.05	0.02	0.03	0.023	0.030	0.094
4–8 m	0.04	0.01	0.01	0.12	0.03	0.02	0.02	0.01	0.01	0.006	0.009	0.000
8–16 m	0.02	0.01	0.01	0.04	0.01	0.01	0.01	0.01	0.01	0.011	0.002	0.017
Above 16 m	0.10	0.03	0.02	0.05	0.02	0.01	0.04	0.03	0.02	0.014	0.010	0.003

### Prospective and Retrospective Analysis

The LTRE analysis estimating the contribution of different matrix types from different habitats and years to the variation in population growth rates suggested no overall significant differences between the matrices (Table 4; Table S2). In the LTRE testing the interaction between the habitat type and year, only one transition matrix (on the middle positions in the last transition period) significantly deviated from the mean matrix showing the lowest population growth rate (Table S2).

**Table 4:** The results of life table response experiment (LTRE) analyses comparing the main effect of the habitat type (position on the slope) on the population growth rate, decomposed into the contributions from single matrix elements, based on matrices from the 3-year dataset.

Transition		Upper part		Middle part		Lower part	
From	To	Contrib.	p	Contrib.	p	Contrib.	p
Above 16 m	Seedlings	**–0.004**	**0.03**	0.000	0.87	**0.004**	**0.03**
Seedling	Up to 0.15 m	0.000	0.96	0.000	0.76	0.000	0.69
Up to 0.15 m	Up to 0.15 m	**0.003**	**0.01**	**–0.002**	**0.01**	**–0.001**	**0.01**
Up to 0.15 m	0.15–0.5 m	**0.002**	**0.01**	**–0.002**	**0.03**	0.000	0.52
0.15–0.5 m	0.15–0.5 m	0.000	0.82	0.000	0.95	0.000	0.69
0.15–0.5 m	0.5–1 m	0.000	0.97	0.000	0.77	0.000	0.68
0.5–1 m	0.5–1 m	0.001	0.32	–0.001	0.42	0.000	0.96
0.5–1 m	1–2 m	0.000	0.85	0.000	0.68	–0.001	0.47
1–2 m	1–2 m	0.001	0.09	–0.001	0.41	–0.001	0.55
1–2 m	2–4 m	0.000	0.89	0.001	0.56	–0.001	0.62
2–4 m	2–4 m	0.001	0.39	0.000	0.90	–0.001	0.40
2–4 m	4–8 m	0.000	0.92	0.001	0.60	–0.001	0.64
4–8 m	4–8 m	0.001	0.59	–0.001	0.53	0.000	0.92
4–8 m	8–16 m	–0.001	0.71	0.001	0.70	0.000	0.86
8–16 m	8–16 m	0.000	0.86	0.000	0.60	0.000	0.70
8–16 m	Above 16 m	–0.001	0.47	0.001	0.22	–0.001	0.55
Above 16 m	Above 16 m	–0.001	0.29	0.000	0.85	0.001	0.30
Overall		0.007	0.39	–0.005	0.62	–0.002	0.78

P indicates the significance of these contributions, determined from permutation tests. Significant values are formatted in bold.

The results of LTRE analysis performed for each matrix element separately indicated significant differences in matrix structure between the matrices. Specifically, the population growth at upper positions was significantly positively driven by the variation in small sapling survival and growth (second and third size categories) and negatively driven by the variation in generative reproduction. This indicates that small sapling survival and growth increases population growth rate at the upper position compared to the other two positions, while, due to low generative reproduction, the overall population growth rate of the upper position is not significantly different from the other positions. On the other hand, the growth of the population at the lower position was significantly positively driven by the variation in generative reproduction and negatively driven by the variation in survival in the second size category. This indicates that the population growth rate at the lower positions is increased thanks to high generative reproduction and decreased due to low sapling survival. Growth in the middle part of the slope was significantly negatively driven by the variation in small sapling survival and growth (second and third size categories) indicating that low levels of small sapling survival and growth caused low population growth rate at this location (Table 4).

Similarly, the LTRE based on data from the 11 transition intervals (with year-specific data on growth and constant seedling natality and mortality) suggested that there were no significant differences in growth rates between populations in different habitat types and years. In this case, there was also no significant interaction detected between the habitat type and year (not shown).

The survival of the oldest trees exhibited the highest elasticity in all years and in all habitat types in both datasets, followed by transitions representing the growth of the trees (i.e., transitioning into larger size category) in all years and in all habitat types in both datasets (Table S3).

## Discussion

Analyses of the effects of different parameters on increment size confirm that size categories are a better determinant of tree growth than age, as suggested, e.g., by Harper [62], justifying the use of size to divide the life cycle of *P. strobus* in the present study. The same pattern can be observed in the data on mortality. In this case, the year of death also has a high predictive power, suggesting strong between-year variations in the mortality of this species. All the other factors with significant effect on length of the increments and mortality, such as locality, habitat type and many interactions, in fact explain very low proportion of total variation. This, however, correspond to various other ecological studies reporting percentage of variance explained in their tables (e.g. [63], [64], in most studies the value is in fact not reported).

According to the matrix models, the investigated populations of *P. strobus* are expected to grow in all habitat types in all transition intervals, despite the declining tendency of population growth observed in the last 11 years. Population growth rates of greater than one in most populations and years have also been reported in other studies on invasive plant species (e.g., [65], [27], [19], [66], [67], and for a summary of matrix analyses of invasive plants, see Ramula et al. [29]). Compared to these studies, the population growth rates recorded for *P. strobus* are much closer to 1 and less variable between years and habitat types. The low variation detected in population growth rates, not only between years but also between habitat types, contrasts with the fact that the different environments differ strongly regarding the yearly trends of temperature, humidity and irradiation (Wild et al. unpubl.). A population growth rate of only slightly above 1, similar to that of *P. strobus*, has been reported for other invasive plant species, but only for certain years or localities [16], [68], [69]. The dynamics of the *P. strobus* populations are also quite similar to the dynamics of long-lived trees under stable environmental conditions [70], [33], [71], [72], [73] and [74] Appendix S1. Long-lived trees are usually characterized by population growth rates just above 1 with low variability as well as a high elasticity of transitions related to stasis, mainly associated with the survival of the largest tress.

In spite of the overall similarity in the population growth rates of *P. strobus* observed in the different habitat types, our results indicate that populations from different habitats vary in the contribution of different vital rates to the observed variation in population growth rates. This suggests that *P. strobus* exhibits different growth strategies in different habitats in the sandstone region, all of which lead to similar population growth rates, which could be the key factor for the successful invasive behavior of this species observed over all environments.

In our dataset, there was also no significant effect of the year identified using LTRE analysis. The effect of the year is non-significant, in spite of the significant effect of the year on both increment length and mortality detected in the univariate analyses. This confirms previous conclusions that significant differences between single plant traits do not necessarily imply significant differences in population growth rates, e.g., [75], [53], [76]. This mismatch between the conclusions regarding single life history traits and overall population dynamics can be attributed to the low elasticity of the most variable matrix elements.

The results of the prospective, elasticity, analysis differ strongly from the results of the retrospective, LTRE, analysis performed using our data, cf. [77]. The results of both types of analyses have previously been used to design management strategies for species, e.g., [78], [16], [79], [80], [81]. While LTRE analysis can identify transitions that actually drive variation in population growth rates, it is elasticity analysis that indicates which transitions should be the target of active management actions [77].

The results of elasticity and LTRE analyses are comparable for the datasets based on 3 or 11 transition intervals, suggesting that the structure of the matrices is the same, irrespective of the period over which the data were collected. This small difference may be because the data on natality and mortality employed in the two datasets were the same, and only the growth transitions were year specific. Natality is known to be the most variable part of the life cycle in many plant species [62] and also mortality of often highly variable between years [38], [39] as shown also in our study. If data on natality and mortality had been available for all 11 years, we might have expected larger differences to be detected between the datasets. The two datasets identified similar amounts of spatial and temporal variation in population growth rates and detected overall similar patterns. They also provided largely comparable results regarding the overall population growth rates. However, in comparison with the 3-year dataset, the 11-year dataset provides additional insights into dynamics over longer time periods and thus enables us to capture possible extreme events. Specifically, the 11-year dataset shows a recent decline in population growth rates. This decline may be interpreted as a slowing down of the invasion process, as most of the study area has already been colonized by this species. However, the long-term data for some size categories suggest that the observed decline may simply represent a fluctuation in the population growth rate and that it may start increasing again at a later stage. No decline in population growth rates over time can be observed in the 3-year dataset, which employed year-specific data on seedling survival and mortality but covers too short a period to reveal any long-term trends. This finding suggests that by collecting simplified long-term data, we obtained different information than by collecting more detailed data over a short time period. Our approach to long-term data collection could be easily used to collect long-term data for other tree species.

The population transition matrices employed in this study assume a lack of density dependence and therefore correspond to most other published matrix transition models e.g., [75], [16], [53], [60], but see Cropper and Loudermilk [82]. The absence of density dependence in population matrix models can be an important drawback when modeling dense stable populations of a species [55]. However, we suggest that this issue is not a major problem in the case of a spreading invasive species that has yet to reach its carrying capacity, such as *P. strobus* in the investigated sandstone areas of the Czech Republic. This effect is mitigated to a degree because results have to be interpreted as describing population growth under current density conditions, and partial density dependence is therefore implicitly incorporated in the transition probabilities, cf. [55], [16].

Density effects could also theoretically affect our results as the trees have different density in different localities and habitat types. This quite high variation in density of *P. strobus* is, however, largely given by the fact that the mature *P. strobus* trees are mostly of plantation origin and they were planted in different manner in different locations. All our experimental plots were, however, located in dense forest stands. The locations with low density of *P. strobus* had thus high density of *P. abies* and/or *P. sylvestris* and possible intra-specific competition between *P. strobus* was at these sites replaced by inter-specific competition. Under the untested assumption that the level of intra- specific and inter-specific competition in this species is similar, the level of density dependence in different experimental plots is thus comparable.

### Conclusions

According to the matrix models, the investigated populations of *P. strobus* are expected to grow in all habitat types in all transition intervals. This finding corresponds to the field observations indicating that the populations of this species are still growing. In agreement with previous studies, the elasticity analysis showed that the survival of the largest categories contributed the most to population growth. In contrast, the LTRE analysis showed that different stages in different habitats contribute the most to the population growth rate. Specifically, generative reproduction is more important at lower positions, and survival is most important for the upper positions. This difference is also observed in the stable stage distribution, which predicts much larger numbers of seedlings at lower positions. These findings indicate that *P. strobus* exhibits different growth strategies in different locations, resulting in similar population growth. In all locations, the population growth rates are above one, indicating that the species is still spreading and has the ability to occupy a wide range of habitats.

## Supporting Information

Figure S1 Increment size for trees (cm) in the category between 4 and 8 m high in the three habitat types.
(TIF)

Figure S2 Effect of year, size category and habitat type on length of the increment (cm) of the trees.
(TIF)

Table S1 Population transition matrices for 3 habitat types over 3 transition intervals.
(DOC)

Table S2 Results of life-table response experiment analysis comparing differences between matrices in the three habitat types (position on the slope) and three transition intervals and their interaction using the mean matrices constructed for habitat type. Positive or negative numbers at the column contribution indicate if the given matrix, positively or negatively contributes to overall population growth rate. P indicates significance of these contributions. Significant values (p≤0.05) are bold.
(DOC)

Table S3 Elasticity and its 95% confidence interval of single transition elements of matrices in the different habitat types summarized over years in the 3 year dataset.
(DOC)

Text S1 Methods of the LTRE analysis.
(DOC)
