# Supplementary figures and images for: Variability in the Contribution of Different Life Stages to Population Growth as a Key Factor in the Invasion Success of Pinus strobus
Source: PLoS One. 2013 Feb 28;8(2):e56953. doi: 10.1371/journal.pone.0056953 (PMC3585251; doi:10.1371/journal.pone.0056953)

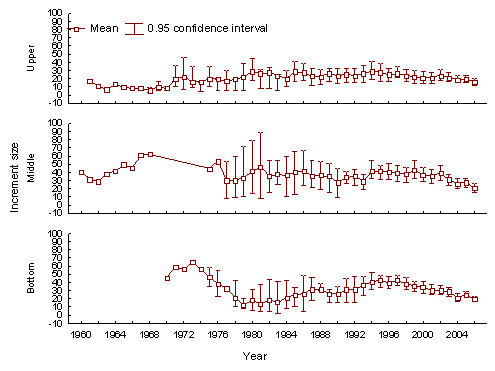

Supplement: Figure S1 — Increment size for trees (cm) in the category between 4 and 8 m high in the three habitat types. (TIF) [file pone.0056953.s001.tif]
