# Supplementary material for: Variability in the Contribution of Different Life Stages to Population Growth as a Key Factor in the Invasion Success of Pinus strobus
Source: PLoS One. 2013 Feb 28;8(2):e56953. doi: 10.1371/journal.pone.0056953 (PMC3585251; doi:10.1371/journal.pone.0056953)
Supplement: Table S1 — Population transition matrices for 3 habitat types over 3 transition intervals. (DOC) [file pone.0056953.s003.doc]

Table S4. Population transition matrices for 3 habitat types over 3 transition intervals.

|  |  | 2004-2005 | | | | | | | | | 2005-2006 | | | | | | | | | 2006-2007 | | | | | | | | |
| --- | --- | --- | --- | --- | --- | --- | --- | --- | --- | --- | --- | --- | --- | --- | --- | --- | --- | --- | --- | --- | --- | --- | --- | --- | --- | --- | --- | --- |
|  |  | Seedling | <0.15 m | <0.5 m | <1 m | <2 m | <4 m | <8 m | <16 m | >16 m | Seedling | <0.15 m | <0.5 m | <1 m | <2 m | <4 m | <8 m | <16 m | >16 m | Seedling | <0.15 m | <0.5 m | <1 m | <2 m | <4 m | <8 m | <16 m | >16 m |
| Upper | Seedling | 0 | 0 | 0 | 0 | 0 | 0 | 0 | 0 | 8.28 | 0 | 0 | 0 | 0 | 0 | 0 | 0 | 0 | 5.09 | 0 | 0 | 0 | 0 | 0 | 0 | 0 | 0 | 1.68 |
|  | <0.15 m | 0.40 | 0.65 | 0 | 0 | 0 | 0 | 0 | 0 | 0 | 0.66 | 0.65 | 0 | 0 | 0 | 0 | 0 | 0 | 0 | 0.52 | 0.65 | 0 | 0 | 0 | 0 | 0 | 0 | 0 |
|  | <0.5 m | 0 | 0.06 | 0.84 | 0 | 0 | 0 | 0 | 0 | 0 | 0 | 0.06 | 0.85 | 0 | 0 | 0 | 0 | 0 | 0 | 0 | 0.06 | 0.85 | 0 | 0 | 0 | 0 | 0 | 0 |
|  | <1 m | 0 | 0 | 0.15 | 0.83 | 0 | 0 | 0 | 0 | 0 | 0 | 0 | 0.14 | 0.83 | 0 | 0 | 0 | 0 | 0 | 0 | 0 | 0.13 | 0.84 | 0 | 0 | 0 | 0 | 0 |
|  | <2 m | 0 | 0 | 0 | 0.14 | 0.85 | 0 | 0 | 0 | 0 | 0 | 0 | 0 | 0.15 | 0.84 | 0 | 0 | 0 | 0 | 0 | 0 | 0 | 0.14 | 0.85 | 0 | 0 | 0 | 0 |
|  | <4 m | 0 | 0 | 0 | 0 | 0.11 | 0.90 | 0 | 0 | 0 | 0 | 0 | 0 | 0 | 0.12 | 0.91 | 0 | 0 | 0 | 0 | 0 | 0 | 0 | 0.10 | 0.91 | 0 | 0 | 0 |
|  | <8 m | 0 | 0 | 0 | 0 | 0 | 0.07 | 0.97 | 0 | 0 | 0 | 0 | 0 | 0 | 0 | 0.07 | 0.97 | 0 | 0 | 0 | 0 | 0 | 0 | 0 | 0.07 | 0.97 | 0 | 0 |
|  | <16 m | 0 | 0 | 0 | 0 | 0 | 0 | 0.02 | 0.96 | 0 | 0 | 0 | 0 | 0 | 0 | 0 | 0.02 | 0.95 | 0 | 0 | 0 | 0 | 0 | 0 | 0 | 0.02 | 0.95 | 0 |
|  | >16 m | 0 | 0 | 0 | 0 | 0 | 0 | 0 | 0.03 | 0.986 | 0 | 0 | 0 | 0 | 0 | 0 | 0 | 0.04 | 0.986 | 0 | 0 | 0 | 0 | 0 | 0 | 0 | 0.04 | 0.986 |
| Middle | Seedling | 0 | 0 | 0 | 0 | 0 | 0 | 0 | 0 | 26.6 | 0 | 0 | 0 | 0 | 0 | 0 | 0 | 0 | 26.3 | 0 | 0 | 0 | 0 | 0 | 0 | 0 | 0 | 4.64 |
|  | <0.15 m | 0.40 | 0.29 | 0 | 0 | 0 | 0 | 0 | 0 | 0 | 0.66 | 0.29 | 0 | 0 | 0 | 0 | 0 | 0 | 0 | 0.52 | 0.29 | 0 | 0 | 0 | 0 | 0 | 0 | 0 |
|  | <0.5 m | 0 | 0.03 | 0.84 | 0 | 0 | 0 | 0 | 0 | 0 | 0 | 0.03 | 0.86 | 0 | 0 | 0 | 0 | 0 | 0 | 0 | 0.03 | 0.86 | 0 | 0 | 0 | 0 | 0 | 0 |
|  | <1 m | 0 | 0 | 0.15 | 0.78 | 0 | 0 | 0 | 0 | 0 | 0 | 0 | 0.13 | 0.78 | 0 | 0 | 0 | 0 | 0 | 0 | 0 | 0.13 | 0.81 | 0 | 0 | 0 | 0 | 0 |
|  | <2 m | 0 | 0 | 0 | 0.16 | 0.79 | 0 | 0 | 0 | 0 | 0 | 0 | 0 | 0.16 | 0.80 | 0 | 0 | 0 | 0 | 0 | 0 | 0 | 0.13 | 0.80 | 0 | 0 | 0 | 0 |
|  | <4 m | 0 | 0 | 0 | 0 | 0.11 | 0.88 | 0 | 0 | 0 | 0 | 0 | 0 | 0 | 0.11 | 0.88 | 0 | 0 | 0 | 0 | 0 | 0 | 0 | 0.11 | 0.90 | 0 | 0 | 0 |
|  | <8 m | 0 | 0 | 0 | 0 | 0 | 0.09 | 0.96 | 0 | 0 | 0 | 0 | 0 | 0 | 0 | 0.09 | 0.96 | 0 | 0 | 0 | 0 | 0 | 0 | 0 | 0.07 | 0.97 | 0 | 0 |
|  | <16 m | 0 | 0 | 0 | 0 | 0 | 0 | 0.03 | 0.95 | 0 | 0 | 0 | 0 | 0 | 0 | 0 | 0.03 | 0.94 | 0 | 0 | 0 | 0 | 0 | 0 | 0 | 0.03 | 0.95 | 0 |
|  | >16 m | 0 | 0 | 0 | 0 | 0 | 0 | 0 | 0.05 | 0.990 | 0 | 0 | 0 | 0 | 0 | 0 | 0 | 0.05 | 0.990 | 0 | 0 | 0 | 0 | 0 | 0 | 0 | 0.04 | 0.990 |
| Bottom | Seedling | 0 | 0 | 0 | 0 | 0 | 0 | 0 | 0 | 24.5 | 0 | 0 | 0 | 0 | 0 | 0 | 0 | 0 | 24.5 | 0 | 0 | 0 | 0 | 0 | 0 | 0 | 0 | 54.2 |
|  | <0.15 m | 0.40 | 0.41 | 0 | 0 | 0 | 0 | 0 | 0 | 0 | 0.66 | 0.41 | 0 | 0 | 0 | 0 | 0 | 0 | 0 | 0.52 | 0.41 | 0 | 0 | 0 | 0 | 0 | 0 | 0 |
|  | <0.5 m | 0 | 0.05 | 0.84 | 0 | 0 | 0 | 0 | 0 | 0 | 0 | 0.05 | 0.83 | 0 | 0 | 0 | 0 | 0 | 0 | 0 | 0.05 | 0.85 | 0 | 0 | 0 | 0 | 0 | 0 |
|  | <1 m | 0 | 0 | 0.15 | 0.77 | 0 | 0 | 0 | 0 | 0 | 0 | 0 | 0.16 | 0.78 | 0 | 0 | 0 | 0 | 0 | 0 | 0 | 0.14 | 0.79 | 0 | 0 | 0 | 0 | 0 |
|  | <2 m | 0 | 0 | 0 | 0.14 | 0.78 | 0 | 0 | 0 | 0 | 0 | 0 | 0 | 0.13 | 0.79 | 0 | 0 | 0 | 0 | 0 | 0 | 0 | 0.12 | 0.79 | 0 | 0 | 0 | 0 |
|  | <4 m | 0 | 0 | 0 | 0 | 0.10 | 0.83 | 0 | 0 | 0 | 0 | 0 | 0 | 0 | 0.09 | 0.83 | 0 | 0 | 0 | 0 | 0 | 0 | 0 | 0.08 | 0.84 | 0 | 0 | 0 |
|  | <8 m | 0 | 0 | 0 | 0 | 0 | 0.08 | 0.97 | 0 | 0 | 0 | 0 | 0 | 0 | 0 | 0.07 | 0.97 | 0 | 0 | 0 | 0 | 0 | 0 | 0 | 0.06 | 0.98 | 0 | 0 |
|  | <16 m | 0 | 0 | 0 | 0 | 0 | 0 | 0.03 | 0.94 | 0 | 0 | 0 | 0 | 0 | 0 | 0 | 0.03 | 0.94 | 0 | 0 | 0 | 0 | 0 | 0 | 0 | 0.02 | 0.95 | 0 |
|  | >16 m | 0 | 0 | 0 | 0 | 0 | 0 | 0 | 0.05 | 0.997 | 0 | 0 | 0 | 0 | 0 | 0 | 0 | 0.04 | 0.997 | 0 | 0 | 0 | 0 | 0 | 0 | 0 | 0.03 | 0.997 |
