# Supplementary material for: Variability in the Contribution of Different Life Stages to Population Growth as a Key Factor in the Invasion Success of Pinus strobus
Source: PLoS One. 2013 Feb 28;8(2):e56953. doi: 10.1371/journal.pone.0056953 (PMC3585251; doi:10.1371/journal.pone.0056953)
Supplement: Table S2 — Results of life-table response experiment analysis comparing differences between matrices in the three habitat types (position on the slope) and three transition intervals and their interaction using the mean matrices constructed for habitat type. Positive or negative numbers at the column contribution indicate if the given matrix, positively or negatively contributes to overall population growth rate. P indicates significance of these contributions. Significant values (p≤0.05) are bold. (DOC) [file pone.0056953.s004.doc]

Table S5. Results of life-table response experiment analysis comparing differences between matrices in the three habitat types (position on the slope) and three transition intervals and their interaction using the mean matrices constructed for habitat type. Positive or negative numbers at the column contribution indicate if the given matrix, positively or negatively contributes to overall population growth rate. P indicates significance of these contributions. Significant values (p ≤ 0.05) are bold.

|  | Contrib. | P |
| --- | --- | --- |
| Upper | 0.007 | 0.39 |
| Middle | -0.005 | 0.62 |
| Bottom | -0.002 | 0.78 |
| 2005 | -0.003 | 0.33 |
| 2006 | 0.001 | 0.66 |
| 2007 | 0.002 | 0.56 |
| Upper 2005 | -0.002 | 0.75 |
| Upper 2006 | 0.008 | 0.3 |
| Upper 2007 | -0.003 | 0.68 |
| Middle 2005 | -0.003 | 0.75 |
| Middle 2006 | 0.009 | 0.23 |
| Middle 2007 | **-0.015** | **0.05** |
| Bottom 2005 | -0.004 | 0.58 |
| Bottom 2006 | -0.001 | 0.88 |
| Bottom 2007 | 0.011 | 0.16 |
