# Supplementary material for: Variability in the Contribution of Different Life Stages to Population Growth as a Key Factor in the Invasion Success of Pinus strobus
Source: PLoS One. 2013 Feb 28;8(2):e56953. doi: 10.1371/journal.pone.0056953 (PMC3585251; doi:10.1371/journal.pone.0056953)
Supplement: Table S3 — Elasticity and its 95% confidence interval of single transition elements of matrices in the different habitat types summarized over years in the 3 year dataset. (DOC) [file pone.0056953.s005.doc]

Table S6. Elasticity and its 95% confidence interval of single transition elements of matrices in the different habitat types summarized over years in the 3 year dataset.

| Transition | | Upper slope | | Middle slope | | Bottom slope | |
| --- | --- | --- | --- | --- | --- | --- | --- |
| From | To | Mean | Conf. int. | Mean | Conf. int. | Mean | Conf. int. |
| Above 16 m | Seedling | 0.052 | (0.047-0.056) | 0.054 | (0.046-0.06) | 0.064 | (0.059-0.067) |
| Seedling | Up to 0.15 m | 0.052 | (0.047-0.056) | 0.054 | (0.046-0.06) | 0.064 | (0.059-0.067) |
| Up to 0.15 m | Up to 0.15 m | 0.073 | (0.062-0.084) | 0.019 | (0.016-0.023) | 0.033 | (0.028-0.038) |
| Up to 0.15 m | 0.15-0.5 m | 0.052 | (0.047-0.056) | 0.054 | (0.046-0.06) | 0.064 | (0.059-0.067) |
| 0.15-0.5 m | 0.15-0.5 m | 0.009 | (0.008-0.011) | 0.011 | (0.009-0.013) | 0.011 | (0.01-0.012) |
| 0.15-0.5 m | 0.5-1 m | 0.052 | (0.047-0.056) | 0.054 | (0.046-0.06) | 0.064 | (0.059-0.067) |
| 0.5-1 m | 0.5-1 m | 0.017 | (0.015-0.02) | 0.016 | (0.013-0.018) | 0.018 | (0.016-0.02) |
| 0.5-1 m | 1-2 m | 0.052 | (0.047-0.056) | 0.054 | (0.046-0.06) | 0.064 | (0.059-0.067) |
| 1-2 m | 1-2 m | 0.010 | (0.008-0.011) | 0.011 | (0.009-0.012) | 0.012 | (0.01-0.013) |
| 1-2 m | 2-4 m | 0.052 | (0.047-0.056) | 0.054 | (0.046-0.06) | 0.064 | (0.059-0.067) |
| 2-4 m | 2-4 m | 0.007 | (0.005-0.008) | 0.008 | (0.006-0.009) | 0.008 | (0.006-0.009) |
| 2-4 m | 4-8 m | 0.052 | (0.047-0.056) | 0.054 | (0.046-0.06) | 0.064 | (0.059-0.067) |
| 4-8 m | 4-8 m | 0.001 | (0.001-0.002) | 0.002 | (0.002-0.003) | 0.002 | (0.002-0.003) |
| 4-8 m | 8-16 m | 0.052 | (0.047-0.056) | 0.054 | (0.046-0.06) | 0.064 | (0.059-0.067) |
| 8-16 m | 8-16 m | 0.002 | (0-0.022) | 0.003 | (0-0.011) | 0.003 | (0-0.029) |
| 8-16 m | Above 16 m | 0.052 | (0.047-0.056) | 0.054 | (0.046-0.06) | 0.064 | (0.059-0.067) |
| Above 16 m | Above 16 m | 0.414 | (0.368-0.459) | 0.440 | (0.386-0.526) | 0.337 | (0.307-0.377) |
