# Supplementary material for: Variability in the Contribution of Different Life Stages to Population Growth as a Key Factor in the Invasion Success of Pinus strobus
Source: PLoS One. 2013 Feb 28;8(2):e56953. doi: 10.1371/journal.pone.0056953 (PMC3585251; doi:10.1371/journal.pone.0056953)
Supplement: Text S1 — Methods of the LTRE analysis. (DOC) [file pone.0056953.s006.doc]

Text S2. Methods of the LTRE analysis.

To perform the LTRE analysis, we followed the approach described in Caswell [50], and calculated a mean matrix (A). The mean matrix is a matrix where each matrix element is a mean of corresponding elements in all the matrices used in the analysis. Population growth rate was expressed as follows:

λ^py^ = λ.. + α^p^ + β^y^ + αβ ^py^, (2)

where λ.. is the population growth rate of the mean matrix A. and the effects of α^p^ is the main effect of habitat type, β^y^ is the main effect of year and αβ ^py^ is the interaction between habitat type and year. The contribution of the transition a_ij_ to the effect of habitat type, year and their interaction on population growth can be expressed as follows:

α^p^ = ∑ (a_ij_^p^. + a_ij_..)s_ij_, (3)

β^y^ = ∑ (a_ij_^.y^ + a_ij_..)s_ij_, (4)

αβ ^py^ = ∑ (a_ij_^py^ + a_ij_..)s_ij_ - α^p^ - β^y^ (5), where s_ij_ is the sensitivity of the ij^th^ transition.

The LTRE analysis indicates the contribution of each life-cycle transition to differences between habitat types. Important life-cycle transitions are those with large absolute values of contributions at some factor level. Analogous to analysis of variance, the mean of the treatment effects is zero. The LTRE analysis included also a significance test estimating significance of the differences between the mean matrices and the different types of matrices. The calculations were done using a MATLAB script developed in a previous study [Münzbergová et al. 2007]. The significance estimation was based on 10 000 permutations in each case.

Reference: Münzbergová, Z. 2007. Population dynamics of diploid and hexaploid populations of a perennial herb. Ann Bot-London 100:259-1270.
